# Class III Malocclusion Surgical-Orthodontic Treatment

**DOI:** 10.1155/2014/868390

**Published:** 2014-11-06

**Authors:** Bruna Alves Furquim, Karina Maria Salvatore de Freitas, Guilherme Janson, Luis Fernando Simoneti, Marcos Roberto de Freitas, Daniel Salvatore de Freitas

**Affiliations:** Bauru Dental School, University of São Paulo, Al. Octávio Pinheiro Brisolla, 9-75, 17012-901 Bauru, SP, Brazil

## Abstract

The aim of the present case report is to describe the orthodontic-surgical treatment of a 17-year-and-9-month-old female patient with a Class III malocclusion, poor facial esthetics, and mandibular and chin protrusion. She had significant anteroposterior and transverse discrepancies, a concave profile, and strained lip closure. Intraorally, she had a negative overjet of 5 mm and an overbite of 5 mm. The treatment objectives were to correct the malocclusion, and facial esthetic and also return the correct function. The surgical procedures included a Le Fort I osteotomy for expansion, advancement, impaction, and rotation of the maxilla to correct the occlusal plane inclination. There was 2 mm of impaction of the anterior portion of the maxilla and 5 mm of extrusion in the posterior region. A bilateral sagittal split osteotomy was performed in order to allow counterclockwise rotation of the mandible and anterior projection of the chin, accompanying the maxillary occlusal plane. Rigid internal fixation was used without any intermaxillary fixation. It was concluded that these procedures were very effective in producing a pleasing facial esthetic result, showing stability 7 years posttreatment.

## 1. Introduction

Occlusal discrepancies and moderate and severe dental and facial deformities in adults usually require treatment combined with orthodontics and orthognathic surgery to achieve optimal, stable, functional, and esthetic results. The basic objectives of orthodontics and orthognathic surgery are to meet patient' complaints, establish optimal functional outcomes, and promote good esthetic results. To achieve this, the orthodontist and the surgeon must be able to correctly diagnose dental and skeletal deformities and establish an appropriate treatment plan for that patient [1]. Class III malocclusion is a difficult anomaly to understand. Studies conducted to identify the etiologic features of Class III malocclusion showed that the deformity is not restricted to the jaws but involves the total craniofacial complex [2, 3]. Most subjects with Class III malocclusions have combinations of skeletal and dentoalveolar components [4]. The factors contributing to the anomaly are complex.

In skeletal Class III cases, it may be difficult to achieve an excellent occlusal outcome only with orthodontic treatment and to maintain a stable posttreatment occlusion [5]. There are three main treatment options for skeletal Class III malocclusion: growth modification, dentoalveolar compensation, and orthognathic surgery. Growth modification should be initiated before the pubertal growth spurt; afterwards, only two options are possible [6]. Thus, treatment of skeletal Class III malocclusion in an adult requires orthognathic surgery combined with conventional orthodontic treatment aiming to improve self-esteem and achieve normal occlusion and improvement of facial esthetics [7, 8]. Proffit et al. [9] found that psychological rather than morphologic characteristics probably were the major reason on whether or not an individual decided to accept surgery. Bell et al. [10] also pointed out that the decision of surgery was mainly related to patients' self-perception.

Surgical treatment of Class III malocclusion includes, in most cases, mandibular retrusion, maxillary protrusion, or a combination of both [6]. Mandibular clockwise rotation can also provide the same result as mandibular retrusion, when increase of lower anterior face height is allowed. Therefore, the objective of this paper is to present a case of a skeletal Class III malocclusion orthodontic surgically treated. Although the problem appeared to be a protruded mandible, the orthognathic surgery included a counterclockwise rotation of the mandibular occlusal plane with advancement of pogonion, segmentation of the maxilla with advancement and expansion, and surgical protrusion of the chin. The pros and cons of these procedures are discussed.

## 2. Case Presentation

**Figure 1:**
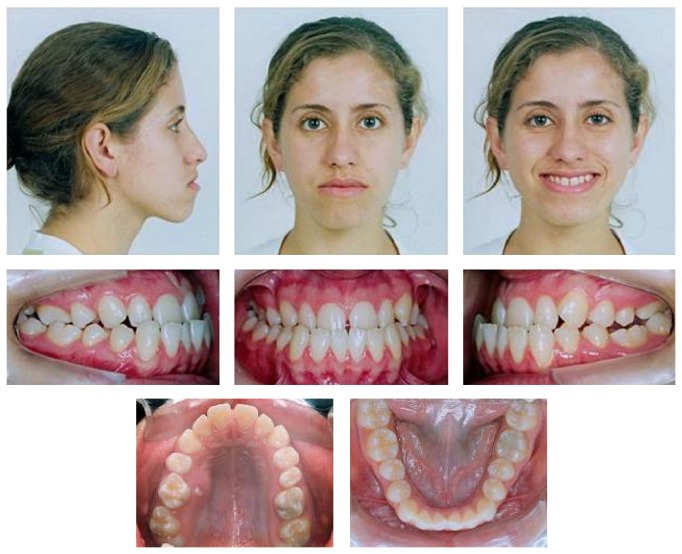
Pretreatment extraoral and intraoral photographs.

**Figure 2:**
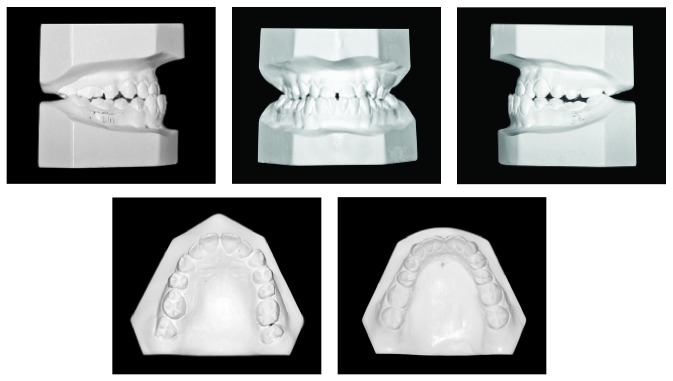
Pretreatment dental casts.

**Figure 3:**
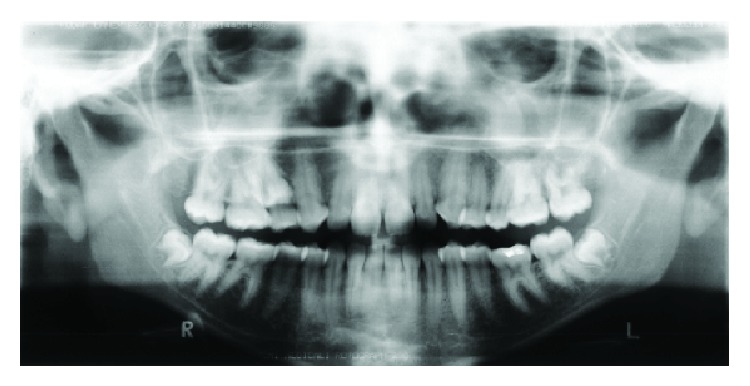
Pretreatment panoramic radiograph.

A 17-year-and-9-month-old female, who had menarche at 12, came for orthodontic treatment to the private orthodontic office with the chief complaints of poor facial esthetics associated with mandibular and chin protrusion. Clinically, the patient did not present an acceptable facial balance; the soft tissue profile was concave, with strained lip closure. Intraoral and dental cast examinations demonstrated severe Class III molar and canine relationships, (molar 3/4-cusp Class III on the right side and full-cusp Class III on the left side). Space analysis was performed at the start of the treatment to assess the space; however, no discrepancy was found. Crowding analysis was also performed and a negative discrepancy model of 1 mm was found. The maxillary arch was constricted, with anterior and posterior crossbites, the mandibular arch showed slight anterior crowding, and there was a 5 mm of negative overjet and an overbite of 3 mm. Maxillary and mandibular midlines were coincident with the facial midline. The mandibular third molars and the maxillary right second premolar were impacted (Figures 1, 2, 3, and 11 and Table 1).

## 3. Treatment Objectives

The primary treatment objectives were to correct the Class III canine relationship, overjet, and overbite and especially to improve facial esthetics. The complementary treatment objectives were to establish good functional and stable occlusion and to improve the smile characteristics and dental esthetics.

## 4. Treatment Alternatives

One of the treatment options consisted of extraction of the impacted maxillary right second premolar and the mandibular third molars, followed by surgically assisted rapid maxillary expansion to improve the constricted maxillary arch and extraction of the mandibular first premolars. The use of mini-implants as mandibular anchorage would help in reducing the overjet and correct the slight mandibular anterior crowding, resulting in Classes I and III molar relationships on the right and left sides, respectively, and Class I canine relationships.

The other treatment alternative would be extraction of the impacted right second maxillary premolar and the mandibular third molars, followed by surgically assisted rapid maxillary expansion to improve the constricted maxillary arch and mandibular setback.

The third option included extraction of the impacted right second maxillary premolar and the mandibular third molars followed by surgically assisted segmented maxillary expansion associated with advancement and impaction and counterclockwise rotation of the mandible with pogonion advancement and surgical chin protrusion.

The treatment options were presented to the patient and discussed. Because the patient was very concerned with improving her facial esthetics and the maxilla appeared to be retruded, the third option was chosen because it would be performed in only one surgical intervention.

## 5. Treatment Progress

**Figure 4:**
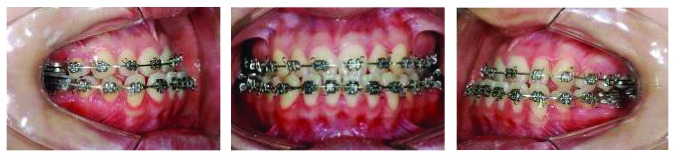
Presurgical intraoral photographs.

Preoperative orthodontic preparation was conducted with preadjusted 0.022 × 0.030-inch fixed appliances. After extraction of the right second maxillary premolar and the mandibular third molars, leveling and alignment with Nitinol and stainless steel archwires of progressively increasing thickness were performed. After leveling and alignment, 0.021 × 0.025-inch stainless steel rectangular archwires were placed in the maxillary and mandibular arches in preparation for surgery. Kobayashi hooks were then attached to all brackets in both arches to allow placement of 1/4-inch intermaxillary elastics after surgery (Figure 4). The presurgical orthodontic phase lasted 11 months.

**Figure 5:**
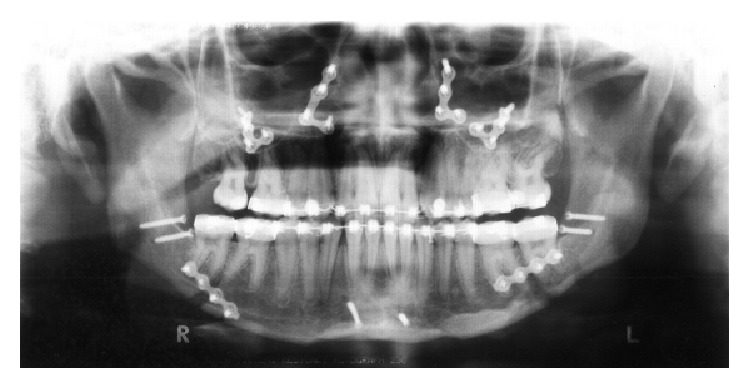
Postsurgical panoramic radiograph.

The surgical procedures included a Le Fort I osteotomy for expansion, advancement, impaction, and rotation of the maxilla to correct the occlusal plane inclination. There was 2 mm of impaction of the anterior portion of the maxilla and 5 mm of extrusion in the posterior region. A bilateral sagittal split osteotomy was performed in order to allow counterclockwise rotation, accompanying the maxillary occlusal plane. Horizontal osteotomy of the mandibular symphysis was performed. This genioplasty was performed due to the impact that other facial osteotomies planned caused on the prominence of the chin. Rigid internal fixation with titanium plates and screws of 2 mm system was used without any intermaxillary fixation (Figure 5).

During the postoperative period, sensory and objective tests were performed to monitor the expected losses of sensitivity. Bianchini, 1995, states that the sensation impairment in an orthognathic surgery can occur partially (paresthesia or hypoesthesia) or completely (anesthesia), caused by microdamage or nerve compressions. These mentioned alterations can recover spontaneously; however, if a complete lesion occurs in the alveolar inferior nerve, a definitive anesthesia is determined. This sensitivity deficit may occur in the mental region, mandibular dentoalveolar region, and lower lip, when mandibular osteotomies are executed [11]. Sensory tests were performed using synthetic brushes of various calibers, whereas thermal tests were assessed using needles of various gauges in the lower lip region bilaterally. Return of normal sensations was observed in the fourth month after the surgery. Considering the patient's reports of improvement in sensations, no special treatment was necessary. After seven years, the neurosensitivity was normal, mouth opening was 40 mm, and mandibular functions were totally normal.

After orthognathic surgery, orthodontic finishing was performed in order to obtain better teeth interdigitation. The patient was instructed to wear vertical intermaxillary elastics for 20 hours a day during 45 days and then gradually reduce the wear time. Occlusal equilibration was performed after appliance removal to refine the interocclusal contacts. A maxillary Hawley retainer and a fixed canine to canine mandibular retainer were placed. Total treatment time was 20 months (Figure 8).

**Figure 6:**
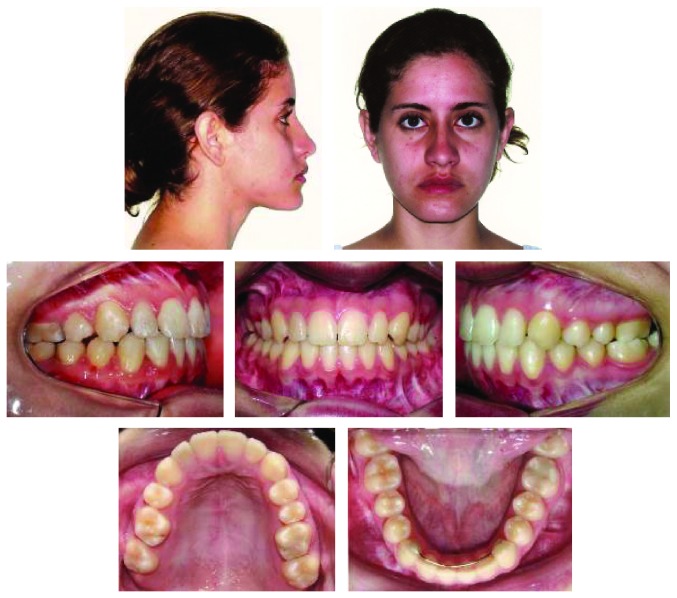
Posttreatment extraoral and intraoral photographs.

**Figure 7:**
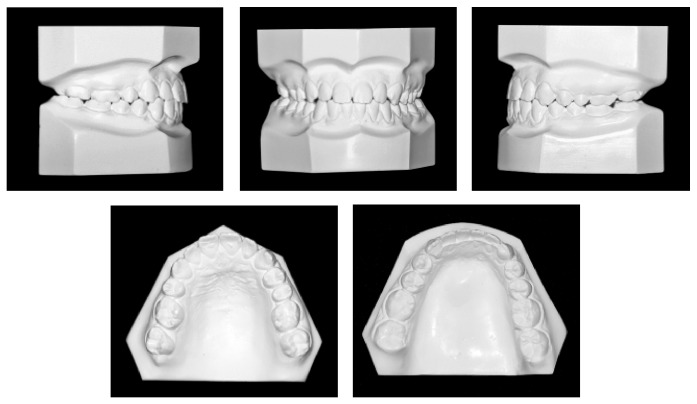
Posttreatment dental casts.

**Figure 8:**
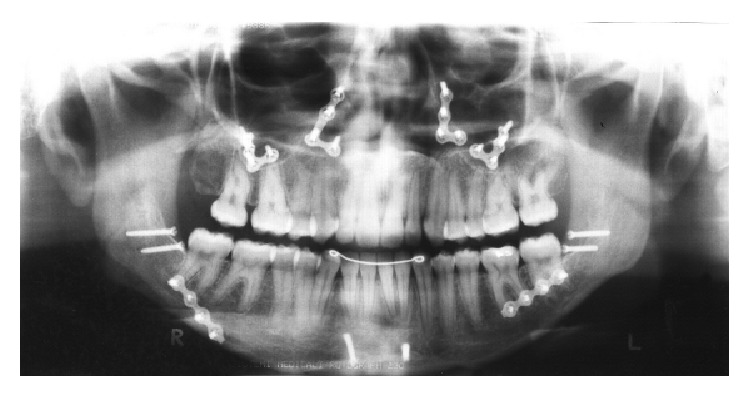
Posttreatment panoramic radiograph.

The facial posttreatment photographs show improvement in the facial profile. The patient was satisfied with his teeth, profile, and smile line. The final occlusion shows Class I canine relationship on both sides and normal overjet and overbite (Figures 6, 7, and 11).

The maxillary incisors were labially tipped and slightly protruded, the mandibular incisors were lingually tipped and retruded, and there was reduction in facial convexity (Table 1). Root resorption was minimal (Figure 10). Transversal increases of 4 mm were observed in the intercanine region (49 mm to 53 mm) and in the intermolar region (63 mm to 68 mm). Superimposition of the cephalometric tracings shows the maxillary advancement and the mandibular counterclockwise rotation, projecting the chin anteriorly (Figure 11).

**Figure 9:**
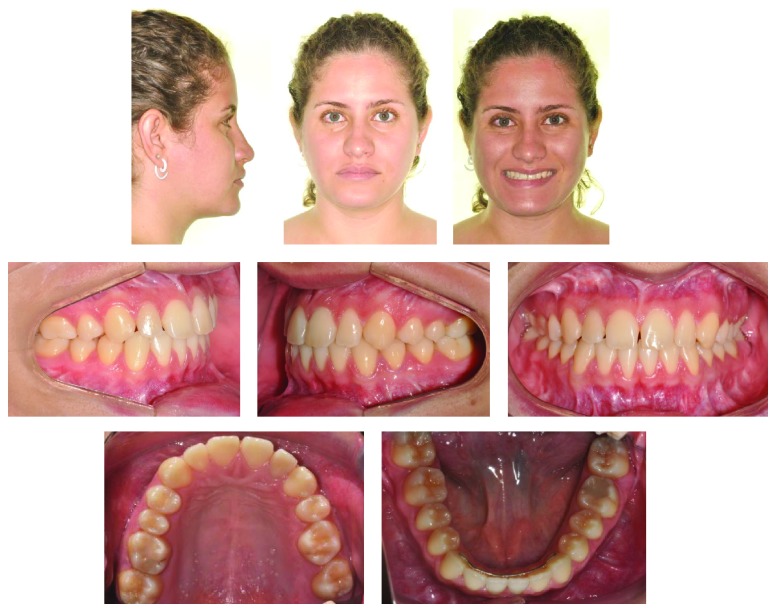
Posttreatment extraoral and intraoral photographs after 7 years.

**Figure 10:**
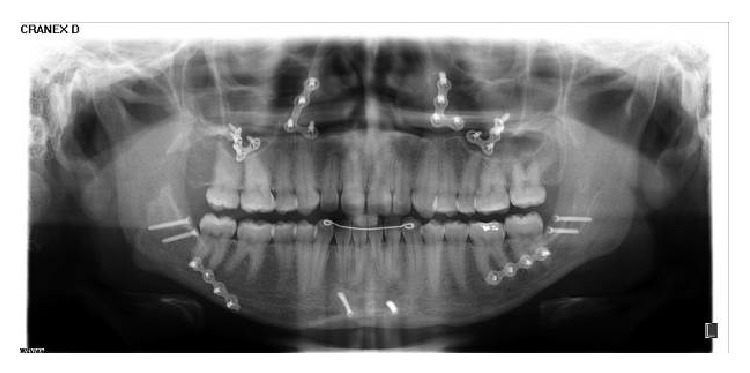
7-year posttreatment panoramic radiograph.

**Figure 11:**
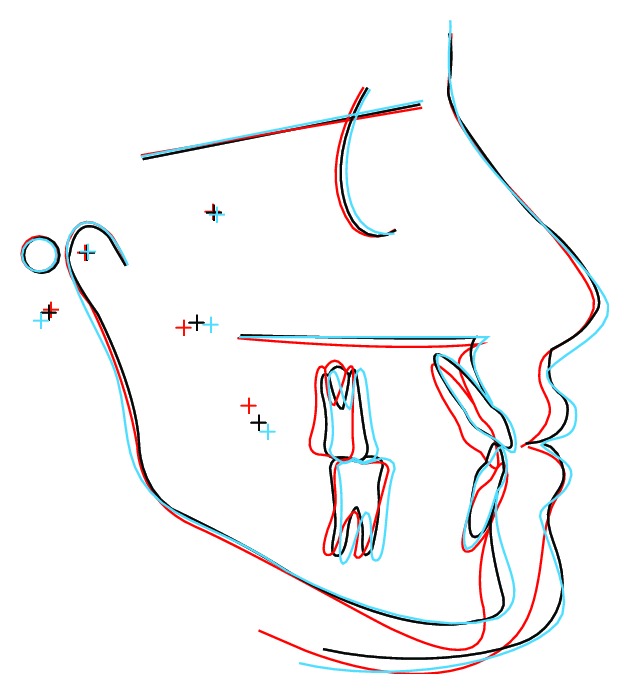
Pretreatment, postsurgical, and 7-year posttreatment cephalometric superimposition (S-N).

The case remained stable 7 years after treatment (Figures 10 and 11 and Table 1); the maxillary and mandibular incisors had a slight increasing in their positive buccolingual inclination. The soft profile and the pogonion had mild advancement 7 years after treatment, probably due to a late growth of the patient's mandible. We can see only a small diastema between the maxillary central incisors that did not bother the patient, so no action was taken (Figure 9).

## 6. Discussion

Correction of maxillary constriction is an important part of the surgical-orthodontic treatment plan. Segmental Le Fort I osteotomy is considered an effective procedure to correct transverse deficiencies. While surgically assisted rapid maxillary expansion (SARME) is performed as the first step of a 2-step approach, segmental Le Fort I is performed concomitantly with the osteotomy. Because time is required for expansion and a postoperative healing period is necessary after SARME, the entire surgical orthodontic treatment time can be prolonged [12]. During treatment planning, some factors to decide between SARME and segmental Le Fort I should be considered: presence of other maxillary problems, magnitude of width deficiency, and stability.

Regarding stability, it is known that maxillary expansion is the most unstable movement in orthognathic surgery after the first postoperative year [13]. Comparisons between techniques of rapid maxillary expansion surgically assisted (two surgical times) and segmented maxillary osteotomy (one surgical time) found that there are no long-term differences. Studies show slightly higher stability when the surgery is performed in a single procedure [12, 14].

It was necessary to perform a maxillary Le Fort I osteotomy, with maxillary segmentation to allow expansion, advancement, and impaction of the maxilla, due to maxillary constriction and posterior and anterior crossbites. However, this procedure should be avoided when a great amount of maxillary expansion is needed, because the palatal tissue thickness does not allow large immediate expansion [15]. Surgery was performed in only one surgical intervention, because it decreases the overall treatment time and the expansion movement is better controlled due to the rigid internal fixation, which increases stability of the surgical results [16–18].

The maxilla was impacted 2 mm in the anterior region to correct the great exposure of the incisors, because maxillary advancement and the surgical access (healing retraction) increase exposure of the maxillary incisors, by changes in upper lip posture. There was also 5 mm of extrusion in the posterior region of the maxilla. However, this subtle impaction was performed because the patient has an increased vertical dimension, and if it was not performed the patient would have an even more vertical profile.

With the surgical repositioning of the mandible for the correction of a prognathic mandible, the technique for the surgical correction of dentofacial deformities has developed into a well-defined science and a fascinating art form. Bilateral sagittal ramus osteotomy is currently the most popular surgical procedure for the correction of dentofacial deformities involving the mandible [19]. Bilateral sagittal split osteotomy was performed to provide counterclockwise rotation of the mandible and chin advancement to enhance dental, skeletal, and soft tissue relationships. The advancement of the chin can be used to improve almost any skeletal abnormality. The technique is primarily used only for esthetic reasons. Moreover, its use is independent of patient care with the appearance of this area of the face. Often, the surgeon has to draw the patient's attention to the need for genioplasty when other facial osteotomies are planned because of the impact that these osteotomies have on the prominence of the chin [1].

Due to the impact of the planned facial osteotomies, the mandible was rotated counterclockwise, and it was necessary to move the chin forward to correct facial height and improve the esthetics of face.

According to the literature, maxillary advancement is the second surgical procedure more associated with relapses in maxillofacial surgery, so that the possibility of relapses of 2 to 4 mm which occurs is 20% or less. An acceptable stability in combined maxillary and mandibular surgical procedures is obtained when rigid internal fixation is used. Three surgical procedures are susceptible to relapses of 2 to 4 mm in 40 to 50% of the cases: the setback of the mandible, the inferior maxillary repositioning, and the maxillary expansion. The movement direction of the surgical procedures, the type of fixation, and the surgical technique can affect the stability of orthognathic surgery [20]. Stability has improved with the use of stable internal fixation, once it accelerates bone repair, allows immediate mandibular functions, avoids complications from maxillomandibular lock, and facilitates oral hygiene and feeding [21].

Another study evaluated the stability of maxilla superior repositioning using Le Fort I osteotomy in various time intervals. A total of 61 patients were assessed and all of them had at least 2 mm of incisors or molars intrusion. It was observed that skeletal or tooth movement of 2 mm or more occurred in approximately 20% of the patients. During the first 6 weeks after surgery, maxilla showed a strong tendency to move up in nonstable patients. Posterior and anterior maxillary regions tended to be vertically stable in 90% and 80% of patients, respectively. Horizontally, the maxilla was stable in 80% of cases. The changes occurred were related to a move back of the maxillary anterior region when the jaw was surgically advanced. After the first six weeks, the maxillary posterior region was vertically stable in all patients; however, in 20% of them, the cephalometric points of maxillary anterior region moved downward, in opposite direction of the movement which occurred during the surgical procedure. No evidence was found that the amount of presurgical orthodontic movement of incisors, the multiple segmentation of the maxilla in surgery, the presence or absence of mentoplasty and suspension wires, and the number of surgical procedures constitute risk factors for stability. No statistically significant correlation was found between direction of surgical movement and direction of postsurgical movement [22]. The stability in orthognathic surgery has improved with the use of stable internal fixation, since it accelerates bone repair, allows immediate restoration of function, and decreases complications of maxillomandibular lock, favoring acceptance to treatment and facilitating oral hygiene and nutrition patient [21].

Immediately after orthognathic surgery, vertical intermaxillary elastics were introduced to obtain better teeth interdigitation. The patient was instructed to wear the elastics for 20 hours a day during 45 days and then gradually reduce the wear time.

The combined surgical-orthodontic treatment of this case led to a significant facial, dental, and functional improvement. The dental relationship achieved was good. Facially, vertical balance and harmony were obtained and this is perhaps the most important goal achieved, because it was the patient's chief concern.

Skeletal relapses arising from orthognathic surgery occur in the first months after surgery [23]. Most of the soft tissues changes occur one year after surgery, but changes may occur up to 5 years after surgery [24]. The case presented showed no skeletal relapse 7 years posttreatment. A small interincisors diastema in the maxillary arch was observed, but it did not bother the patient, so no action was taken, since the patient discarded retreatment or an esthetic restoration to close the diastema.

In summary, the treatment of dentofacial deformities of young patients that finished craniofacial growth is complex, especially when transversal and sagittal discrepancies exist, requiring orthodontic and orthognathic surgery to achieve stable, functional, and esthetic results. Skeletal Class III malocclusion treatment is difficult; however, an orthodontic-surgical approach for the correction of this alteration has wide acceptance among patients. Orthodontic camouflage of this malocclusion requires a detailed assessment of patient's face. When esthetics is compromised, only an orthodontic treatment is not enough. In these cases, it is necessary to combine orthodontics and orthognathic surgery to meet the patient's complaints and provide better functional and esthetic results. In the present case, the surgical counterclockwise rotation was very effective in producing a pleasing facial esthetic result. Despite the first impression that the case needed mandibular setback, the counterclockwise rotation resulted in an unusual advancement of pogonion, projecting the chin anteriorly, accompanying the maxillary occlusal plane. This protocol showed good occlusal and esthetic results, showing stability 7 years posttreatment.

## Figures and Tables

**Table 1:** Pretreatment and posttreatment cephalometric status measurement.

Variables	Pretreatment	Posttreatment	7-years posttreatment
Maxillary component			
SNA (°)	88.7	89.6	89.9
A-N Perp (mm)	7.5	8.3	8.5

Mandibular component			
SNB (°)	87.9	88.3	90.0
P-N Perp (mm)	12.9	14.4	16.1
P-NB	−0.4	1.0	2.3

Maxillomandibular relationship			
ANB (°)	0.8	1.3	−0.1
NAP (°)	2.1	1.6	1.0

Facial growth pattern			
SNGoGn (°)	34.7	30.5	29.9
SN.Gn	64.7	62.6	61.5

Maxillary dentoalveolar component			
1.NA (°)	23.8	27.3	28.8
1-NA (mm)	4.8	4.9	6.1

Mandibular dentoalveolar component			
1.NB (°)	29.6	22.9	24.0
1-NB (mm)	7.7	3.9	5.0
IMPA (°)	86.9	84.0	86.2

Dental relationships			
INTERINCISAL (°)	125.8	128.0	126.7

Soft tissue component			
UPPER LIP to S (mm)	−1.6	−1.8	0.0
LOWER LIP to S (mm)	3.6	−0.8	−0.1
